# Decision-making about gene therapy in transfusion dependent thalassemia

**DOI:** 10.1186/s12887-022-03598-3

**Published:** 2022-09-09

**Authors:** Maa-Ohui Quarmyne, Diana Ross, Cynthia Sinha, Nitya Bakshi, Jeanne Boudreaux, Lakshmanan Krishnamurti

**Affiliations:** 1 Center for Cancer and Blood Disorder, Phoenix Children’s Hospital, 1919 E Thomas Road, Phoenix, AZ 85286 USA; 2 Aflac Cancer and Blood Disorders Center, Department of Pediatrics, Emory University, 1405 Clifton Road NE, Atlanta, GA 30322 USA; 3 Aflac Cancer and Blood Disorders Center, Children’s Healthcare of Atlanta, 1405 Clifton Road NE, Atlanta, GA 30322 USA; 4 Yale Pediatric Hematology Oncology and Bone Marrow Transplant, Yale School of Medicine, Yale University, 35 Park Street, CT 06511 New Haven, USA

**Keywords:** Decision-making, Transfusion dependent thalassemia, Gene therapy, Qualitative

## Abstract

**Background:**

Hematopoietic stem cell transplantation (HSCT) is a treatment option with curative intent for patients with transfusion dependent thalassemia (TDT) but its application is limited by the lack of suitable donors and acceptability due to the related morbidity/mortality. Transplantation of autologous genetically modified hematopoietic cells, gene therapy (GT) is emerging as a promising treatment option for TDT as it eliminates graft versus host disease (GVHD) and need for immunosuppression. Early results of GT suggest that many, but not all patients achieve transfusion independence after the procedure. There is little information about the acceptability of GT in patients with TDT*.* We sought to examine patient/family knowledge about GT in TDT and to examine factors that influence decision-making about this therapy.

**Methods:**

Parents of children with TDT and adults with TDT were who provided informed consent underwent semi-structured interviews to understand patient/family knowledge and decision-making regarding GT in TDT. Transcribed interviews were coded and the data was examined for emerging themes using a combination of thematic and content analysis.

**Results:**

Twenty-five study participants with mean age of 38Y (17—52Y) including eight adults living with TDT, and 17 parents of children with TDT underwent semi-structured qualitative interviews. Participant responses coalesced around broad themes related to knowledge of GT, motivating/deterring factors and outcomes. Study participants expressed a desire for ‘cure’ from thalassemia including transfusion independence, chelation reduction and improved quality of life as motivators for considering GT. Insufficient knowledge about the process, long-term outcomes, safety, and side effects as well as the potential for death/failure of the procedure were deterrents for the consideration GT. Reduction in frequency of transfusions, even without elimination of transfusions was an acceptable outcome of GT for most participants. Participant choice for preferred treatment modality was split between indefinitely continuing transfusions which was familiar to them versus GT which was unfamiliar, and with an uncertain outcome. None of the participants had a matched sibling donor; alternate donor HSCT was the least preferred option in this group.

**Conclusion:**

There is tempered excitement about GT in patients/families with TDT with a general willingness to accept transfusions reduction as the outcome.

**Supplementary Information:**

The online version contains supplementary material available at 10.1186/s12887-022-03598-3.

## Background

Transfusion dependent thalassemia (TDT) is a major public health problem with substantial economic, personal and health care burdens worldwide. An estimated 56,000 babies are born annually with major thalassemia, of which 30,000 require transfusions to survive [1]. In developing countries, where the large majority of patients with TDT reside, chronic transfusion therapy is often not feasible. Even among those with access to safe and adequate transfusion, only a small minority have access to adequate chelation therapy to prevent or treat transfusional iron overload and less than 40% of transfused patients receive adequate chelation [1, 2].

Hematopoietic stem cell therapy (HSCT) offers a curative treatment option with event free and overall survival rates of 80% and 90% respectively for HLA-identical sibling donor transplants [3]. However, less than 25% of patients are likely to find suitable matched sibling donor for transplant [4, 5]. Alternate donor HSCT, is associated with lower overall survival and higher risks of graft versus host disease (GVHD) [3, 6–8]. The emergence of gene therapy (GT) involving the transplantation of genetically modified autologous hematopoietic stem cells has the potential to eliminate the need for an allogeneic donor and the risk of GVHD, and greatly expand the applicability of HSCT for TDT [9–11].

Qualitative studies seeking to determine the perspective of patients living with sickle cell disease (SCD) and their families suggest that they seek curative options for a variety of reasons including the concern about diminishing quality of life, a hope for their child to have a more “normal” life or a recent severe SCD related complication [12]. These studies also suggest that families consider GT as a less toxic alternative to HSCT but remain concerned about the side effects of chemotherapy, the potential for developing a subsequent malignancy and the risk of infertility. Interestingly they also expressed moral concerns about GT which conflict with their personal value system (‘playing God’) and equitable access to these newer therapies [12–15]. In Hemophilia A & B, adeno-associated virus (AAV) liver-directed GT techniques have been employed with the goal of decreasing prophylactic factor replacement and bleeding symptoms in patients with severe hemophilia. While these treatments, do not involve myeloablative chemotherapy as for SCD and TDT, patient perspectives about GT in hemophilia have also provided insights into patient perspectives about novel GT-based treatments that offer the potential for decreasing chronic treatments and disease related morbidity. In a qualitative study, investigating patient perspectives about GT in patients with Hemophilia A and B, Overbeeke et al. report that most patients have positive attitudes toward GT. Factors that were most important in decision-making in this study were annual bleeding rate, factor level, uncertainty of long-term risks, impact on daily life and the possibility of stopping prophylaxis. While patients expressed concerns about the uncertainty of long-term safety of this therapy, they were less concerned about long-term efficacy as even short-term relief from factor administration was considered beneficial and patients knew they could fall back on factor administration if needed [16]. There are currently no published data examining patient knowledge and perspectives about GT in thalassemia. This study sought to examine patient/family knowledge about GT in TDT, evaluate motivators and deterrents for GT in patients/families with TDT and to assess what would be considered to be an acceptable outcome of GT.

## Methods

Patients or parents of patients < 18 years with TDT were recruited for this study from the Comprehensive Thalassemia Center at Children’s Healthcare of Atlanta (CHOA), a thalassemia family camp organized by CHOA and from attendees of the 2018 Cooley’s Anemia Foundation (CAF) Conference in Atlanta. The study utilized a purposive sampling approach i.e., a selective, non-random sampling technique. This iterative, flexible approach to participant enrollment allowed for a diverse socio-demographic cohort.

The study eligibility criteria included (i) patients living with TDT or parents of children with TDT (ii) fluency in English (iii) ability to provide informed consent. The study was approved by the Emory University Institutional Review Board. Patients were excluded from study participation if they could not speak English.

An interview guide was developed after review of the literature on patient and caregiver perspective on GT for hemoglobinopathies/thalassemia and was reviewed and validated by the research team with expertise in clinical management of TDT (MQ, JB, NB), BMT and GT (LK), and qualitative research (NB, CS, NB, and LK). The initial section of the interview guide focused on patient demographics and thalassemia history, followed by knowledge of GT, open ended questions about factors that could influence a participant’s decision-making about GT. The final section of the interview guide focused on desired outcomes, and it included a mix of open-ended and closed-ended questions. Questions regarding personal preference for outcomes following GT particularly with regards to transfusion independence versus reduction, acceptable hemoglobin levels following GT, preferred modality of treatment (BMT vs transfusions vs GT) and overall impression of GT, were closed-ended (Supplement 1). The semi-structured interviews were conducted in person or over the phone after informed consent had been obtained by one investigator, MOQ. Each interview lasted about 30–60 min. Participant preferences on what constitutes acceptable outcomes were captured explicitly and without influence by any investigator’s likely biases. The interviewer (MOQ), also used several strategies to mitigate the possibility of social desirability bias [17] by using open ended questions, probing answers and prefacing sensitive questions. Each interview was audio recorded, transcribed verbatim and checked for accuracy before being subjected to qualitative analysis.

An a priori list of defined codes was developed (deductive codes) based on literature review and data from prior qualitative research studies about patient/family choices and decision-making about treatment options in sickle cell disease, conducted by members of the research team (LK, NB, CS, DR). Additional codes (inductive codes) were added to capture the themes that emerged from each interview. MOQ, DR, and CS reviewed codes after initial coding of 10 transcripts to resolve discrepancies in the coding process and ensure consistency in the coding process. MOQ coded all interviews; DR and CS each independently coded 25% of transcripts and these were compared to coding done by MOQ to ensure consistency. Thematic saturation was reached when no new themes emerged in the last few interviews and there was depth of understanding of patient knowledge and decision making about GT. Studies assessing code development and development in qualitative research have demonstrated that up to twelve interviews may be adequate for code saturation, however a larger sample size (as in this study with twenty-five in depth interviews), allows for the development of richness and understanding of the themes raised [18, 19].

A combination of thematic and content analysis approach was used in data analysis. Thematic and content analysis in qualitative research are approaches to data analysis that allow researchers to organize data and capture themes in qualitative data sets, to provide a narrative understanding of participant experiences. While content analysis focuses on establishing categories in data and describing the frequencies of categories identified, thematic analysis on the other hand identifies patterns of meaning in datasets [20]. The study analysis was carried out using NVivo Release 1.3. Descriptive statistics, where applicable were calculated for demographic data. The Consolidated criteria for reporting qualitative research (COREQ) [21] guidelines were followed in reporting these research findings.

## Results

### Study population

Twenty-five participants were interviewed for the study but one interview was not successfully recorded so 24 transcripts were available for data analysis. The majority of study participants (68%) were parents of children with TDT, with children ranging in age from 2-17Y (mean 8Y) at the time of the study. Only one parent per child was interviewed for the study. Eight participants (32%) were patients aged 17-52Y living with TDT. The study participants were relatively highly educated. The youngest participant was in high school; all other participants had some college education and 36% of participants had postgraduate or professional degrees. Table 1 shows additional demographic information on the study population. Seventy-two percent of interviews were over the phone and lasted an average of 44 (range 26–58) minutes compared to 37 (range 28–58) minutes for in-person interviews. About 30% of participants interviewed had previously established rapport with the interviewer, mostly as patients.

**Table 1:** Demographics of study participants

	All participants (*N* = 25)	Parents (N 17)	Patients (N 8)
Sex			
• Female		11 (64.7%)	5 (62.5%)
Age (years)	36 (17–52)	40.5 (33 – 51)	28.5 (17 – 52)
Ethnicity			
• African-American / Black	1 (4%)	1	0
• Asian	10 (40%)	6	4
• Caucasian	14 (56%)	10	4
Educational Level			
• High School	1 (4%)	0	1
• Some college/Associate’s Degree	3 (12%)	2	1
• Bachelor’s degree	12 (48%)	9	3
• Post graduate/Professional	9 (36%)	6	3
US Regions			
• Midwest (OH)	2 (8%)		
• Southeast (AL, FL, GA, LA)	21 (84%)		
• Northeast (MA, NJ)	2 (8%)		
Religion			
• Buddhist	1 (4%)	1	0
• Christian	13 (52%)	10	3
• Hindu	1 (4%)	1	0
• Muslim	5 (20%)	4	1
• None	5 (20%)	1	4
Number of children with TDT	1 (Range 1 – 4)	1 (Range 1 – 4)	n/a
Age of children	8 (Range 2 – 17)	8 (Range 2 – 17)	n/a
Adoptive vs Biological Parents			
• Adoptive	9 (53%)	9 (53%)	n/a
• Biological	8 (47%)	8 (47%)	n/a
Fathers vs Mothers			
• Fathers	6 (35%)	6 (35%)	n/a
•Mothers	11 (65%)	11 (65%)	n/a

### Thalassemia history and complications

All participants interviewed described initiation of transfusions in childhood, thus all participants had experience with iron overload and chelation therapy, either as patients themselves or parents of children who needed chelation. Sixty-three percent described endocrine co-morbidities (delayed puberty, fertility, growth, adrenal suppression); 54% described gastrointestinal complications (nausea, ulcers, abdominal pain) and 50% of participants reported splenomegaly/splenectomy. Other complications described by participants include alloimmunization, renal dysfunction, pain, cardiac problems, visual changes and bony changes.

### Knowledge of gene therapy

All participants interviewed were aware of gene therapy. Most participants also received information about gene therapy as part of the interview process, after asking about basic knowledge questions, but before delving into factors that would influence decision-making about GT. About 80% of participants were aware that the process involved the use of chemotherapy but lacked knowledge about specific techniques such as gene addition or gene editing. 75% of the participants mentioned transfusion independence or reduction in transfusions as possible outcomes of GT. Some participants made comments that reflected their misinformation about GT for TDT including statements about use of radiation in the preparative regimen, the need for ‘less’ chemotherapy compared to BMT and statements about GT being cheaper than BMT.

### Decision-making about gene therapy

#### Motivating factors

Participants were favorably disposed towards GT. The key motivating factors for the consideration of GT were (i) desire for transfusion independence or a ‘cure’ from thalassemia (ii) improved quality of life (iii) financial factors (iv) decreased healthcare utilization and (v) decreased iron overload and the burden of chelation (Table 2). About half of participants described the desire for ‘cure’ or transfusion independence as a motivating factor for GT. A third of participants also described other aspects of healthcare utilization including reduced need for imaging studies and provider visits as motivating factors for considering GT (Table 2).

**Table 2:** Motivating factors for gene therapy

	Key Quotes
Motivators	
Transfusion independence	*‘Well, primary motivating factor for me would probably be to be uh, just transfusion independent so, so I don't have to deal with all the medical bills and the uh, stresses of uh, going to the doctor so much. And I don't know… just think it'd be nice to live my life without thalassemia’* [Patient] *‘I say his wellbeing, where he doesn’t have to do this anymore. He doesn’t have to rely on a system to take care of him and he’ll be able to take care of himself because this is a huge system that helps him to stay alive’* [Parent] *‘I’m uh, been doing this over 50 years and it would be nice to retire. You know, if I put in my time and it would be nice to somewhat retire my uh, efforts, you know, my transfusions, and uh, regimen and at the very least, if it were a couple of times a year that would be uh, you know, reduce the amount of times that I’m having to go to uh, get that done, would be a nice switch’* [Patient]
Healthcare Utilization	*‘it would be nice to not uh, um, occupy so much time in the ….you know doctor’s office and getting treatments and being able to spend more time with my family’* [Patient] *‘if I have less frequent visits in a year with my son once he’s cured or his frequency of coming into the hospital for transfusion is reduced then that would also be a great thing for me’* [Parent]
Iron overload and chelation	*‘our main motivator would be that, eventually, hopefully, she wouldn’t have to take the chelators’* [parent] *‘but chelation is certainly an um… a daily uh… on its own schedule. I don’t know the right word for it, but it, fairly minor compared to a blood transfusion, but it’s something that has to be dealt with everyday versus once every 2 to 4 weeks, so that’s definitely a factor’* [parent]
QOL	*‘If you can get a child to become transfusion-free, then it opens up a whole other world that they otherwise wouldn’t have had. You know, he can, if this works, he can go to college and not have to be pinned down to only going to a college where there’s a nearby place for him to get blood. He won’t have to worry about how he would handle, you know, six months overseas while he’s in college as, you know, trying to do something cool like other kids do because he’s not sure how insurance and all that’s going to work in a foreign country’* [parent] *As a parent I am kind of um, stuck to uh, to some of the roles or some of the positions or some of the cities where I can get her uh, the transfusion done ……….., so I can’t explore a lot of career options for myself, uhm, because of uh, her medical condition.* [parent]
Socioeconomic	*‘I have really good insurance, so it pays for his….. But after he’s 26, he’ll have to foot the bill for this his self, and I don’t know what that looks like for him.* [parent]

Participants described how thalassemia and its treatment permeated every aspect of their life, including interruptions to school, employment and vacations. This sentiment was expressed by both patients living with thalassemia and parents of children with thalassemia. Some participants described taking jobs that were less favorable for professional advancement but which had better insurance options, or to be closer to cities with comprehensive thalassemia care. Parents described how thalassemia and its treatment influenced college choices for their children, potentially limiting their child’s college experience for opportunities such as studying abroad. Other lifestyle choices impacted by thalassemia include the need for scheduling travel around transfusions. Both adults living with thalassemia and parents noted that a ‘cure’ for thalassemia could greatly impact their quality of life.

Economic or financial considerations were brought up by about 40% of patients, as an important consideration. Participants talked about the current cost of healthcare and insurance coverage, raising concerns about the potential cost of GT and whether it would be affordable when it became commercially available. While all the patients interviewed had health insurance for care, some adult patients were concerned about continued medical care should they lose their current job with its medical benefits. Some parents also expressed concerns about continued insurance coverage for their children as independent young adults and their ability to continue to have access to care. However, about 20% of participants, all of them parents, mentioned that economic and insurance costs were not a major factor in their decision making.

Some participants also reported that they would be being motivated to pursue GT because if it was recommended by their treating hematologist. Preference for a treatment that did not include the use of an allogeneic donor for HSCT or risk for GVHD also came up as motivating factors for GT.

GT was generally perceived as a ‘safer’, ‘less risky’ or ‘better’ option, compared to BMT.

‘I don’t remember all the specifics; it seems like the process of going through bone marrow transplant is a little more risky than some of the newer gene therapy’ [parent].

‘we’ll like do some research, do some more research about gene therapy and see what I find, but um, to me right now right now that one stacks better in theory because you would be using your own cells.’ [patient].

Some participants stated that living with thalassemia was inherently associated with long-term complications thus rationalizing those risks versus effects of myeloablative chemotherapy and GT.

‘We started also thinking about what all the long-term side effects of a life of thalassemia are and, uh, the risks with the medications, the risks with the blood, um, and just the overall risk of cancer that grows – that grows as they have all the iron in their bodies and things like that. So, we kind of felt like, you know, risk versus gain. We felt like there was probably still quite a bit more gain to gene therapy than there was risk of lifelong thalassemia’ [parent].

### Deterrents

The most prevalent concern about GT was related to the safety of the process and the side effects of chemotherapy. However, participants also accepted chemotherapy conditioning as a necessary price to pay for achieving a cure and not necessarily an unsurmountable barrier to accepting GT.

Some participants expressed concerns about GT being a new therapeutic strategy, with unknown long-term effects. Closely related to this was the concern that they had insufficient knowledge or information about this process. At least a third of patients raised concerns about the possibility of failure of the procedure and 20% of participants noted the possibility of death as deterrents for GT. Other concerns raised included apprehension about ‘genetic manipulation’, ambivalence about trading the known disease process of TDT treatment with transfusion and chelation (which were providing reasonable quality of life), for the uncertain benefits and unknown complications of GT.

The possibility of decrease in disease burden and reduction in out-of-pocket expenses were important motivators for GT for some participants but others were deterred by the possibility that once GT may become unaffordable once licensed. About a quarter of participants reported concerns about the prolonged hospitalization associated with GT potentially posing an unacceptable burden on their family or support systems, significant financial burden and risk to continued employment. Table 3 lists key quotes that supporting these findings.

**Table 3:** Deterrents for gene therapy

	**Key Quotes**
**Deterrents**	
**Chemotherapy**	‘*Um, I don’t know, but, I – I – I don’t know. I just think to put them through something like the chemotherapy and everything involved right now, I don’t know that I would do it right now.*’ [parent] *‘I guess I am a bit concerned about the uh, the uh, process of going through uh, any of the chemotherapy. Um I’ve done my best to keep myself in good shape and I think I’ll be fine, um, but it’s just going through that, I know, it’s an ordeal, so, I mean that’s worrying, but it’s not going to put me off of anything that uh,, you know, could potentially fix my thalassemia’* [patient]
**Genetic manipulation**	‘*it’s growing in your body and it’s your genes that are being edited, maybe there’s some long-term effects, right, maybe your offspring, um, would get you know, would inherit something that’s maybe coming to your gene sequence that wasn’t there before’* [Parent] *‘I don’t even know if we would know that for a generation. You know, of having done this and as those patients have children is when we would really start to find out what’s happening. So um, but I would, I wouldn’t um, I wouldn’t not pursue it because of that you know, if the success rates are great, I wouldn’t not pursue it because there’s a chance that, the editing of the gene can impact the next generation*’ [Parent]
**Trading one disease for another**	*‘could there be a side effect that uh, he currently does not have to deal with….., could the gene therapy introduce a new challenge that he currently doesn’t face… and uh, maybe the answer is no but we would want to become slowly informed about the after effects and side effects of gene therapy before we make any kind of decision’* [Parent]
**Keeping the status quo ‘not rocking the boat’**	‘*chronic transfusions, while they are a uh, disturbance in quality of life, it is a, it’s a known uh, quantity, it’s a, like we know, it’s easy, it’s not simple, but, uh, we know what that probably looks like and we know that there’s little risk associated with it, and that’s just because we did on it for 33 times now I think’* [Parent] ‘*it’s just that they’re very, very hard questions and you know why they’re hard? Because she’s stable right now. If you asked me this four years ago, I would say yes to everything. Cause she was in critical condition, um but now she’s living her normal or normal um, you know transfusions every three weeks is really not that bad for us, and she has a pretty healthy quality of life, so that’s what makes it really hard’* [Parent]
**Possibility of failure**	‘*you have something that is working and it’s not the most ideal but it’s gotten you to this point which is a pretty far point, getting to.., getting to my age, um, and then you’re asked to kind of take a leap of faith that hey, this could work and you could eliminate this great need for this uh, treatment, uh, but then on the other end you know if it doesn’t, do you find yourself in a worse situation’* [Patient]
**Socio-economic**	‘*I guess my biggest concern about gene therapy would be, be err probably the cost ****……****, but how are patients going to be expected to pay for that. What’s that going to look like?*’ [Patient] ‘*I know that it can be a long process, it takes a – at least, um, a few months, um, so I’d be concerned about being out of work for that long. Um, and then if the insurance would cover it, or if I would have to pay all of it out of pocket’*. [Patient]

The perspective of participants on infertility were complex and nuanced. Most adult participants living with TDT expressed concerns about the likelihood of infertility but did not necessarily see it as an ultimate barrier to GT. Two adult participants were however so concerned about the possibility of infertility that they would not consider GT, and were not reassured by the availability of fertility preservation procedures. The other adult participants living with TDT, expressed varied sentiments around future childbearing including the decision not to have children, adoption and fertility preservation options. Parents shared similar sentiments as well however, for some parents, the possibility of infertility was influencing the decision to wait until their children were older to have a more informed discussion with them. Some parents shared the notion of their children avoiding childbearing so as to not transmit the thalassemia mutation, though they acknowledged that this would ultimately be their child’s decision. Further they pointed out that while GT may be associated with impaired fertility, thalassemia itself is associated with infertility (Table 4).

**Table 4:** Fertility and gene therapy

	**Key Quotes**
**Adult patient perspectives**	*‘ …..even if it would leave me infertile, I think I’d be okay with that’*
	*‘…and I think that they recommend that, right, that you, you store um, sperm samples in case you decide on one, and I do want to have children eventually, so that actually, that is a, that is a concern for me but not as strong as the other concerns. Probably like the lowest of my concerns*
	*‘I’m not sure that I would pursue it …., um if I wanted to have kids, right now, um, I mean in the next couple of years for example because it would probably be the last uh, few years where I would be able to do that, so that would be like my priority over gene therapy’*
**Parent Perspectives**	*‘I have to think that the fact that he has a gene, a genetic disorder that will pass down to his children, that might uh, I would think it would probably affect his decision and maybe have him choose to not have biological children, but again, that’s gonna be, that’s gonna be his call when he’s older, so I guess I’m just trying to think towards the future and um, I dunno’*
	*‘I don’t foresee it as being too much different, I think that we still have to worry about it, or if they’re unable to have their own children, that might even be the case today, with the situation today. So I don’t, I don’t think that it’ll be much worse than today, it may be that, maybe, he can have children today but there’s a chance that he’ll pass that you know, beta thalassemia onto his children, versus with gene therapy, he may not be able to have children at all and that’s a, almost a, you know, it’s not the same thing but it’s pretty close to me in my mind’*
	*That’s hard to say, um, simply because it is her decision, but she’s too young to make that decision…………. But that’s kind of hard because even if she keeps with thalassemia, there’s complications if she was to have a child, so don’t really know’*
	*‘Yes, definitely. Especially for our daughter. She, uh, you know, at 7 years old, is absolutely baby-obsessed, and adores babies. So, um, that – that’s something we have talked about, too. That would be a concern. It wouldn’t be a reason we wouldn’t pursue it at all, but, um… We don’t feel like we could… We don’t want to make that decision for her’*

### Outcomes

Over 90% of participants expressed a willingness to accept transfusion reduction of 50–90%, without transfusion independence as the outcome after GT. Only one patient was emphatic about the guarantee of transfusion independence being a condition for pursuing GT, yet, this participant also indicated a preference for GT as the preferred modality of treatment between all options currently available. All participants were also willing to accept mild-moderate anemia as an outcome of GT (Hb levels 9–11). GT and chronic transfusions emerged as the preferred treatment options for participants, with 83% stating a preference for either GT (10/24) or transfusions (10/24). This distribution was similar, for both parents and patients, living with TDT. Only four participants stated a preference for BMT as the preferred option; they included one adult living with TDT and three biological parents. They stated concerns about lack of information about GT (including safety) and hesitancy to adopting a new therapy as reasons for this choice. None of the participants had a matched sibling donor as an option for cure at the time of the interview, this happened by chance and was not deliberate on the part of the investigators. It is possible that the absence of an HLA identical donor impacted participant choices of preferred treatment modality. Table 5 lists key quotes from study participants about their preferred treatment modality.

**Table 5:** Preferred treatment modality

	**Key Quotes**
**Chronic Transfusions**	*If they were equally available? Um, I mean, I think for right now, it would be transfusions, because – just exactly what we’re doing, because their quality of life is so good right now.* [Parent] *Um… I mean, it – if it – if we knew that the gene therapy would work for them, um, being double-zero, I think we would probably be, um, more heavily pursuing that. But, um… I – I guess there’s just still some unknowns in our mind of – of – of the kiddos that have double-zero, or zero zero, or however you say it, um, so… For now, we’re hanging out with the transfusions* [Parent] *So, right now I would probably choose status quo, um, just because I don’t think I have the, I don’t have enough information about gene therapy, I would like to see where the trial goes, before I go down that route, although it is a pretty close second, um, I’m very interested in, in the possibility of gene therapy but I’m not completely there yet, because right now it just isn’t there yet* [Adult patient]
**BMT**	*No, so I would say the only option that I would consider would be a sibling match, or um, someone that comes very close, like 100% or 99% sort of um, match, otherwise, I wouldn’t consider bone marrow, um, it wouldn’t be even be in the table, so then I would wait for gene therapy to prove its success*. [Parent] *Honestly what would have been the best for me would have been when I was like five or six, my parents just were able to…., like I just have a sibling match donor and if I just got a bone marrow transplant, I think that would have probably been the best option. Um, now, I would say probably, I would prefer, I would prefer a successful gene therapy, assuming all my criteria were met, that leaves the other options that you mentioned* [Patient]
**Gene Therapy**	*Um, I would be between um, gene therapy and a transplant, um, but I might want to wait a little bit and see how um, we’ll like do some research, do some more research about gene therapy and see what I find, but um, to me right now right now that one stacks better in theory because you would be using your own cells [Patient]* *No, I would consider gene therapy more because I know that there are lots of other risks of rejection, so gene therapy over BMT* [Parent]

## Discussion

In this qualitative study of decision making about GT in TDT we report the perspective of patients and families regarding knowledge of GT, motivating/deterrent factors and desired outcomes as they consider this therapeutic option. We also observed that while there is general awareness of GT, there was limited knowledge about processes involved, possible outcomes and complications. A similar finding was noted in studies of GT in SCD [13, 15].

Families reported the desire for transfusion independence as a major motivating factor for GT, and ultimately, decreased iron overload/chelation needs, reduced health care burden and improved QOL. Published outcomes from initial GT trials, have indicated the attainment of transfusion independence following GT does not necessarily imply restoration of a normal bone marrow milieu or complete correction of ineffective erythropoiesis, as some degree of ineffective erythropoiesis still exists in patients following GT [9]. Thus, while transfusion independence may not necessarily equate to a complete cure for TDT (i.e., complete correction of ineffective erythropoiesis), the attainment of transfusion independence or significant reduction in transfusions was important to patients/families and generally led to a favorable view of GT in this study population.

Perspectives of participants around the potential side effect of infertility was multifaceted and nuanced. Most participants, both parents and patients living with TDT expressed concerns about the possibility of infertility, but this concern, was not necessarily a ‘deal-breaker’ for the consideration of GT as a therapeutic option. However, for some adult patients and parents, this side effect was influencing the decision hold off pursuing GT. Generally, patients and parents, were encouraged about fertility preservation techniques but this sentiment was not universal. Some participants while acknowledging the side effect of infertility associated with GT also pointed out that TDT, even without GT was also associated with infertility in some patients, thus inferring that GT would not be creating an entirely new problem. Values and attitudes related to fertility and child bearing are greatly influenced by socioeconomic status, cultural experiences and religious beliefs hence the perspective of this study population may not be generalizable to all populations with thalassemia. These findings are at variance with the findings of Strong et al. who reported in focus group study of adult sickle cell patients that the risk of infertility was considered unacceptable and fertility preservation techniques as burdensome [15]. On the other hand, Persaud et al. found that patients with SCD had no concerns about fertility problems, whereas some parents were concerned about treatments that could limit their children’s reproductive viability and the continuation of their family line [14].

Similar to studies in SCD [13, 14], participants in this study had concerns about the cost of GT, affordability and access. An important difference was that participants did not express mistrust of the healthcare system or research enterprise. This may be explained by the racial composition of this study as only 44% of the study population were of minority origin (Table 1), who have traditionally had more mistrust of the investigational therapies and research in the US given historical events and racial inequities in healthcare [22].

Participants in this study perceived BMT to be ‘riskier’ than GT. It is important to note that none of the participants on this study had an available HLA matched related donor at the time of the study which likely influenced the view of BMT as risky or less safe in their particular case. Published outcomes of HSCT in thalassemia report higher event free and overall survival rates for HLA-identical sibling donor transplants and lower risks of graft versus host disease (GVHD, compared to alternate donor HSCT [3, 6–8].

Intriguingly, most participants considered reduction in the frequency of transfusions, even without complete elimination of transfusions to be an acceptable outcome of GT. Employing reflexivity, we were cognizant of the investigators’ bias, that a tradeoff between continuing transfusion and myeloablative chemotherapy with infusion of autologous genetically modified hematopoietic stem cells would only be justifiable if transfusion independence were achieved. We had therefore carefully designed the questions regarding what would constitute an acceptable outcome as close ended questions. While we were surprised that participants were willing to consider reduction of frequency of transfusions without the elimination of transfusion dependence as an acceptable outcome, this response gave us an inkling of the perceived burden of continuing transfusion and chelation indefinitely. These findings are important in light of the observation that in some patients with homozygous β^0^ thalassemia mutations, earlier results from GT studies indicated that it may result in substantial reduction in annualized transfusion volume but not the elimination of transfusion dependence however, refinements in the technology of genetic modification of hematopoietic stem cells indicate that transfusion requirements may ultimately be eliminated even in patients with homozygous β^0^ thalassemia mutations [10, 23]. This study underscores the importance of seeking and clarifying the patient perspective on acceptable outcomes is as a crucial guide to ongoing clinical research.

This study has a number of limitations. The majority of study participants were parents of children with TDT, likely influencing the study results, however, notable comparisons have been made to adult patient versus parent responses, to give more perspective and richness to data collected. Secondly, study participants also had variable understanding about GT prior to their individual interviews, however, patient/families with TDT in the community are making decisions about GT with varied background knowledge and this study, is reflective of that reality. Thirdly, GT was investigational in the US and not FDA approved at the time of the study, and it is likely that factors that would affect decision-making about pursuing a therapeutic option, that is FDA approved may be different from an investigational product. Fourth, none of the study participants had a matched sibling donor available, at the time of the interview. This could have influenced their choice for preferred treatment modality, even though participants were asked to state their preferred choice for treatment, should all options i.e., matched sibling donor, matched unrelated donor, haplo-identical donor, chronic transfusions and gene therapy, be available to them. Finally, in these interviews which were conducted in 2018–2020, we did not probe the issues of cost of GT when it becomes available commercially, or the potential for subsequent malignancy as was subsequently observed in patients with sickle cell disease [24, 25]. It is important to note that while the perspectives of this diverse group of study participants is important in starting to generate an understanding of decision-making about GT in patients and family with TDT, the opinions expressed in this study are not necessarily generalizable to all patients/families with TDT, in the US or across the world. In general, qualitative studies, aim to provide a more personal, in-depth understanding of the individual experience rather than generalizability. Thus, this study provides an in-depth understanding of the knowledge and decision making of thalassemia patients/families in the community interviewed, who are contemplating GT with varied background knowledge.

## Conclusions

This study has provided insights about the perspective of adult patients and parents of children with TDT towards GT. We have identified key motivators and deterrents, knowledge and understanding of GT and what constitutes an acceptable outcome for patients with TDT. The study participants generally view this novel therapeutic modality favorably but the decision making is complex, as participants balance the desire for cure or transfusion reduction/independence versus concerns about side effects of a newer therapy with unknown long-term outcomes. It is imperative that clinicians, and researchers, collaborate with the community in disseminating high-quality information to help families living with TDT make informed shared decisions regarding GT.

## Supplementary Information

**Additional file 1. **

## Data Availability

The data underlying this study consists of individual interview transcripts which cannot be made publicly available due to ethical concerns related to patient confidentiality. Data are available from the Emory University Institutional Review Board for researchers who meet the criteria for access to confidential data. Contact Via 404–712-0720 or IRB@emory.edu.

## Abbreviations

BMT: Bone Marrow Transplant
CAF: Cooley’s Anemia Foundation
COREQ: Consolidated criteria for reporting qualitative research
GT: Gene Therapy
GVHD: Graft Versus Host Disease
Hb: Hemoglobin
HLA: Human Leukocyte Antigen
HSCT: Hematopoietic Stem Cell Transplant
QOL: Quality of Life
SCD: Sickle Cell Disease
TDT: Transfusion Dependent Thalassemia
