# Supplementary material for: Decision-making about gene therapy in transfusion dependent thalassemia
Source: BMC Pediatr. 2022 Sep 9;22:536. doi: 10.1186/s12887-022-03598-3 (PMC9461218; doi:10.1186/s12887-022-03598-3)
Supplement: Supplementary file 1 — Additional file 1. [file 12887_2022_3598_MOESM1_ESM.docx]

**Decision Making about Gene Therapy for Transfusion Dependent Thalassemia**

**Interview Guide**

Aim: To examine patient knowledge about gene therapy and to evaluate prognostic and other factors which influence the decision to pursue gene therapy

PART I

1. Demographics
   - How old is your child? or you? (for patient >18Y)
   - If interviewing parent note if father, mother, both or other guardian
   - Sex
   - What is your State of Residence?
   - What is your Race
     1. African/African American/Black
     2. American Indian or Alaska Native
     3. Asian
     4. Native Hawaiian or Other Pacific Islander
     5. White/Caucasian
     6. Other
   - What is the highest educational level you have achieved?
     1. For patients >/= 18Y
     2. For both parents if child is <18Y
   - What is your Religious Affiliation?
     1. Christian - _____________ (specify)
     2. Jewish
     3. Muslim
     4. Buddhist
     5. Hindu
     6. Atheist/Agnostic
     7. Other
2. Thalassemia & Other Medical History
   - At what age were you (or your child) diagnosed with thalassemia?
   - What type of transfusion dependent thalassemia do you have (i.e. HbH, beta thal, HbE beta thal, etc)
   - At what age did you (or your child) start transfusions?
   - How frequently do you receive transfusions?
   - What complications have you had from your disease and its treatment?
     *(Allow patient to talk about complications then ask specifically about complications listed below if not mentioned)*
     1. Alloimmunization
     2. Iron overload
        - Heart disease (arrhythmias/heart failure)
        - Liver disease
        - Bony changes
        - Enlarged liver/spleen
        - Splenectomy
        - Endocrine
          1. Delayed growth
          2. Delayed puberty (for children >/= 13Y)
          3. Fertility (for adults)
     3. Problems associated with chelation
        - Ophthalmologic
        - Audiology
        - Renal Disease
        - Gastrointestinal
        - Other
   - Do you have any other medical problems?
   - Do you have a family history of transfusion dependent thalassemia?

PART II

1. Gene therapy Knowledge
   - Tell me what you know about gene therapy
     1. What do you know about how patients are prepared for gene therapy?
        - Mention use of cytotoxic/chemotherapy agents and ask if patients/families know about that
        - Mention genetic techniques – briefly mention gene addition vs gene editing techniques and ask if patients know about
     2. What is your knowledge about possible side effects of this therapy?
     3. What is your knowledge about outcomes after this treatment?
        - (*Probe families about knowledges of outcomes - Ability to stop transfusions vs reduction in transfusions vs failure)*
2. Factors affecting a patient/family’s decision to pursue gene therapy or not based on desirable & potential undesirable outcome?
   - What would be a primary motivating factor for you to pursue gene therapy?
     1. What are some of the other desired (good) outcomes which would influence this decision?

*Try to get the family to list at LEAST 3 others, and in order of importance*. *Review these other factors, if not mentioned to see if these are important to families as well as they consider gene therapy*

- - - - *Reduced healthcare utilization – reduced clinic/hospital visits*
      - *Reduction in missed school days*
      - *Reduction in missed days off work*
      - *Other economic factors eg copays etc*
      - *QOL*
  - What is the primary concern that would influence your decision not to pursue gene therapy
    1. What are some of the other undesirable (good) outcomes which would influence this decision.

*Try to get the family to list at LEAST 3 others, and in order of importance*. *Review these other factors, if not mentioned to see if these are important to families as well as they consider gene therapy.*

- *Increased risk of infections (especially peri-transplant)?*
- *Likelihood of infertility? Would fertility preservation options influence this decision?*
- *Possibility of failure*
- *Possible damage to some of your body’s organs?*
- *Possible increased risk of cancer in the future?*
- *Possible limitation in 2^nd^ transplant should that become necessary?*
- *Concern about off target effects*
- *Prolonged hospitalization with BMT, need to take time off work for it & need for follow up visits after BMT.*
  - Additional questions about expected transfusion needs and anemia after gene therapy.
    1. If gene therapy does not result in the ability to discontinue transfusions what degree of reduction in transfusions would you consider acceptable?
    2. If you are able to stop transfusions, but continue to have anemia what would be minimum Hb level that you would consider acceptable (or would a moderate degree of anemia be acceptable?)

1. Gene therapy vs other therapies.

What other options would you consider over gene therapy and why?

(*Ask families to talk about other treatment options they would consider over gene therapy and why. Then specifically ask about the other options to see if families would also consider the other options over gene therapy & why)*

1. Standard care – transfusions
2. ?Matched sibling transplant
3. Matched unrelated donor transplant
4. Haploidentical transplant
5. What are your overall impressions of gene therapy in thalassemia – for you or your child?
   - Definitely pursue it, no reservations
   - Definitely not, based on my current knowledge
   - Maybe if XXXXX condition can be met
   - Maybe but I don’t want to be the first patient
   - I don’t know. I will need more information.
